# Discovering up-regulated VEGF–C expression in swine umbilical vein endothelial cells by classical swine fever virus Shimen

**DOI:** 10.1186/1297-9716-45-48

**Published:** 2014-04-23

**Authors:** Pengbo Ning, Yanming Zhang, Kangkang Guo, Ru Chen, Wulong Liang, Zhi Lin, Helin Li

**Affiliations:** 1 College of Veterinary Medicine, Northwest A&F University, Yangling, Shaanxi 712100, PR China

## Abstract

Infection of domestic swine with the highly virulent Shimen strain of classical swine fever virus causes hemorrhagic lymphadenitis and diffuse hemorrhaging in infected swine. We analyzed patterns of gene expression for CSFV Shimen in swine umbilical vein endothelial cells (SUVECs). Transcription of the vascular endothelial growth factor (VEGF) C gene (VEGF-C) and translation of the corresponding protein were significantly up-regulated in SUVECs. Our findings suggest that VEGF-C is involved in mechanisms of acute infection caused by virulent strains of CSFV.

## Introduction, methods, and results

Classical swine fever (CSF) is a serious swine disease that occurs in most developed countries, including China, which seriously affects the economic development of the pig-breeding industry [1]. Acute CSF is caused by virulent strains of Classical swine fever virus (CSFV), such as the Shimen strain. Symptoms of acute CSF include hemorrhagic lymphadenitis, diffuse hemorrhaging of the skin, kidney and other organs, high fever and depression [2], which acute hemorrhagic lesions would bring high fatality rates [3]. The C strain of CSFV, sharing genotype and probably ancestral viruses with CSFV Shimen, is able to conclude its course of infection but does not cause typical diffuse hemorrhaging [4]. Molecular mechanisms of the host cell pathological response to CSFV Shimen remains poorly understood.

The vascular endothelial growth factor (VEGF) family, including VEGF-A, -B, -C, -D, and placenta growth factor are key regulators of normal and pathological blood vessel growth [5] and vascular permeability [6]. VEGF can induce the formation of small pores, allowing for leakage of small solutes in the thin endothelial cell cytoplasm; these act in a dose-dependent manner, affecting the leakage occurring in these junctions and pores [7,8]. Signaling pathways are implicated in VEGF-induced vascular permeability, while VEGF-mediated intracellular signals moderate distinct activities of VEGF including vascular permeability, cell survival, proliferation, migration, angiogenesis, adult physiology and disease [9]. Plasma or serum VEGF released from circulating cells can transmit signals from the luminal surface of the vascular endothelium [10]. Alternatively, VEGF bound to the extracellular matrix or released from pericytes, stromal cells, or tumor cells can initiate signaling pathways, leading to vascular leaks [11]. When the infection is occurring, VEGF-induced vascular leaking could promote extravasation of proteins such as fibrinogen, which polymerizes to form fibrin in the extracellular space, thereby providing an impenetrable barrier to contain an infection or the malignant growth of cells [12,13].

Although it has been widely reported that vascular lesions are caused the highly virulent strain of CSFV, not enough attention so far has been given to the VEGF family with respect to CSF. Abnormal expression of VEGF family members is closely related to excessive proliferation of differentiating cells, inflammatory exudation, congestion of capillaries, bleeding and other pathological phenomena [14,15]. Here we describe a series of analyses from digital gene expression tag profiling (DGE). We found that VEGF-C was involved in the infection of swine umbilical vein endothelial cells (SUVECs) by CSFV Shimen.

We obtained immortalized SUVECs as previously described [16]. The CSFV Shimen and C strains were obtained from the Control Institute of Veterinary Bioproducts and Pharmaceuticals (Beijing, China). SUVECs were cultured in 25-cm^2^ tissue culture flasks at a density of 2 × 10^7^ cells per flask. The Shimen and C strains of CSFV were added to cultures at an MOI of 10 TCID50 per cell when SUVECs were 70–80% confluent, respectively. After a 1-h incubation at 37 °C/5% CO_2_, medium was aspirated and fresh medium containing 2% fetal calf serum (FCS) was added. Cultures were incubated for a further 72 h at 37 °C/5% CO_2_. High resolution melt (HRM) curve analysis described by Ning et al. [17] was conducted to determine proliferation of CSFV. Total RNA was isolated from CSFV-infected SUVECs and controls using Trizol reagent (Invitrogen, Carlsbad, CA, USA) according to the manufacturer’s instructions. RNA yields were determined by measuring the absorbance of samples at 260 nm using a Nanodrop ND-2000 (Nanodrop Technologies, Wilmington, DE, USA).

A DGE analysis based on Solexa sequencing was designed to investigate molecular changes in SUVECs following infection with CSFV Shimen. The CSFV C strain was used to obtain differentially expressed genes in SUVECs compared to those expressed during a CSFV Shimen infection. Cluster and Java Treeview were used to perform cluster analyses of gene expression patterns to determine similar gene expression patterns [18].

An ABC-ELISA kit, to monitor levels of VEGF-C activation, was obtained from Shanghai Westang Bio-Tech Co., Ltd (Shanghai, China) and used according to the manufacturer’s instructions. Cell culture supernatants were harvested by centrifugation, and samples (100 μL) of supernatants and standards were seeded into 96-well plates and allowed to incubate at 37 °C for 2 h. After washing five times, biotinylated antibody (100 μL) was added to each well and allowed to incubate at 37 °C for 60 min. Enzyme-labeled antibody (100 μL) was then added to wells and allowed to incubate at 37 °C for 30 min. Plates were washed and color developed using TMB solution; after 15 min the enzyme reaction was stopped by adding stop solution. The absorbance at 450 nm in each well was measured using a microplate reader (Multiskan FC, Thermo, Waltham, MA, USA). We presented values as the mean ± the standard error of the mean (SEM).

Total RNA from cells was prepared from CSFV-infected cells and control samples as described above. In quantitative polymerase chain reaction (qPCR) assays, specific oligonucleotide primers were used to determine expression levels of VEGF-C (5′-AAA CAA ACT TTT CCC CAA CTC G-3′ and 5′-CAC GGT CGT CTG TAA CAA CTG C-3′) and β-actin (5′-CAA GGA CCT CTA CGC CAA CAC-3′ and 5′-TGG AGG CGC GAT GAT CTT-3′). Reactions were performed on a Bio-Rad iQ5 system with SYBR Premix Ex Taq II (TaKaRa, Dalian, China). We synthesized cDNA using the Transcriptor First Strand cDNA Synthesis Kit (TaKaRa, Dalian, China), according to the manufacturer’s instruction. Each cDNA was detected in triplicate, after which the average threshold cycle (Ct) was calculated per sample. The 2^–∆∆Ct^ method was used to calculate the relative expression levels in samples.

Whole-cell lysates from SUVECs at various time points after infection were prepared with RIPA Lysis Buffer (Beyotime Biotech. CO., China) according to the manufacturer’s protocol. Total protein concentration in samples was determined using a protein assay kit (Bio-Rad, Hercules, CA, USA) with bovine serum albumin as a standard. The whole-cell lysate extracts were diluted in sample buffer and boiled for 5 min. A portion (20 μg) of each extract was resolved by 10% sodium dodecyl sulfate polyacrylamide gel electrophoresis (SDS–PAGE) and blotted onto nitrocellulose (NC) membranes (Stratagene, La Jolla, CA, USA) with a semidry transfer cell (Bio-Rad Laboratories, Hercules, CA, USA). Membranes were blocked for 2 h at room temperature in TBST blocking buffer (20 mM Tris–HCl pH 7.4, 150 mM NaCl, 0.1% Tween 20) containing 5% skimmed milk powder to prevent nonspecific binding and then incubated with specific antibodies raised against VEGF-C (Cell Signaling Technology, Beverly, MA, USA) or β-actin at room temperature for 2 h. Membranes were washed three times with TBST and then incubated with HRP-conjugated secondary antibodies (1:2000 dilution) for 2 h at room temperature. Immunoreactive bands were visualized using an enhanced chemiluminescence system (ChemiDoc™ XRS, Bio-Rad, Hercules, CA, USA).

Statistical analyses were conducted using SPSS 16.0 (SPSS Inc., Chicago, IL, USA), with a *P*-value less than 0.05 considered significant.

**Figure 1:**
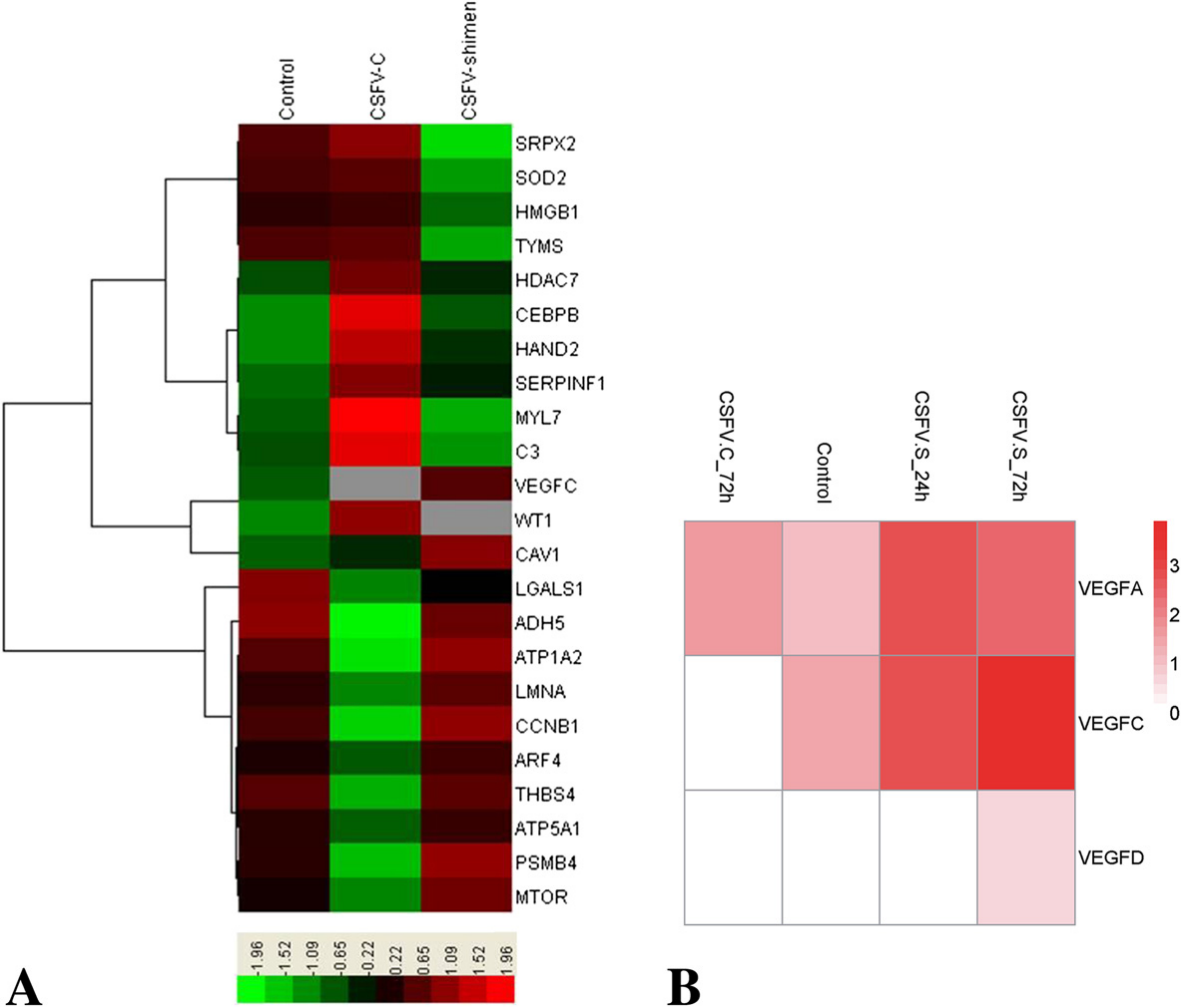
**DGE of SUVECs reveals clusters of regulated genes enriched in the blood circulation system. A**. Clusters display that VEGF-C is a key gene correlated with CSFV Shimen infection to comprise with CSFV C controls and negative controls (mock-infected cells). **B**. Representative heat maps are shown of differentially expressed VEGF family gene clusters along with corresponding CSFV Shimen and C infection.

We used DGE analysis to show differential expression of host genes during CSFV Shimen and CSFV C infections after 72 h. Infection with CSFV Shimen triggered different gene responses compared with CSFV C infection. We identified clusters of key genes that were affected in SUVECs following CSFV Shimen and CSFV C infection (Figure 1A); a significant gene identified was VEGF-C. Genes of the VEGF family clustered together and appeared to be regulated by CSFV Shimen. At 24 hours post-infection (hpi) with CSFV Shimen, we observed that expression levels of VEGF family genes increased over time, with VEGF-C showing the most marked increase (Figure 1B). Significant up-regulated expression of VEGF-C was not observed in SUVECs infected with CSFV C.

**Figure 2:**
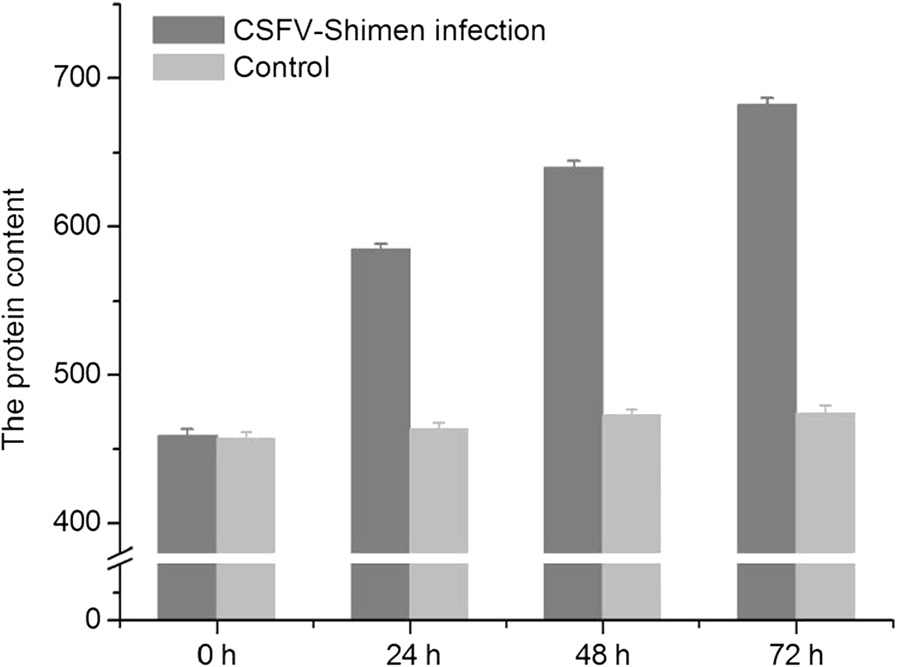
**ELISA analysis of VEGF-C protein levels for SUVECs infected with CSFV Shimen or controls.** SUVECs were infected in the absence or presence of CSFV Shimen, and cell culture supernatants at 0, 24, 48 and 72 h postinfection were harvested to examine levels of VEGF-C activation. Values are means ± the SEM from triplicate wells. Controls, mock-infected cells.

Using ELISA assays we observed a time-dependent increase in VEGF-C expression in CSFV Shimen-infected cells at 72 hpi (Figure 2). At 0, 24, 48, and 72 hpi, expression of VEGF was significantly increased (*P* < 0.05), at 459.02 ± 4.60, 584.68 ± 3.92, 640.07 ± 4.57, and 682.10 ± 4.60 pg/mL, respectively. In addition, VEGF levels were not significantly increased in control cells at the corresponding time points (457.56 ± 3.73, 463.63 ± 4.31, 472.72 ± 3.67, and 474.00 ± 5.17 pg/mL, respectively).

**Figure 3:**
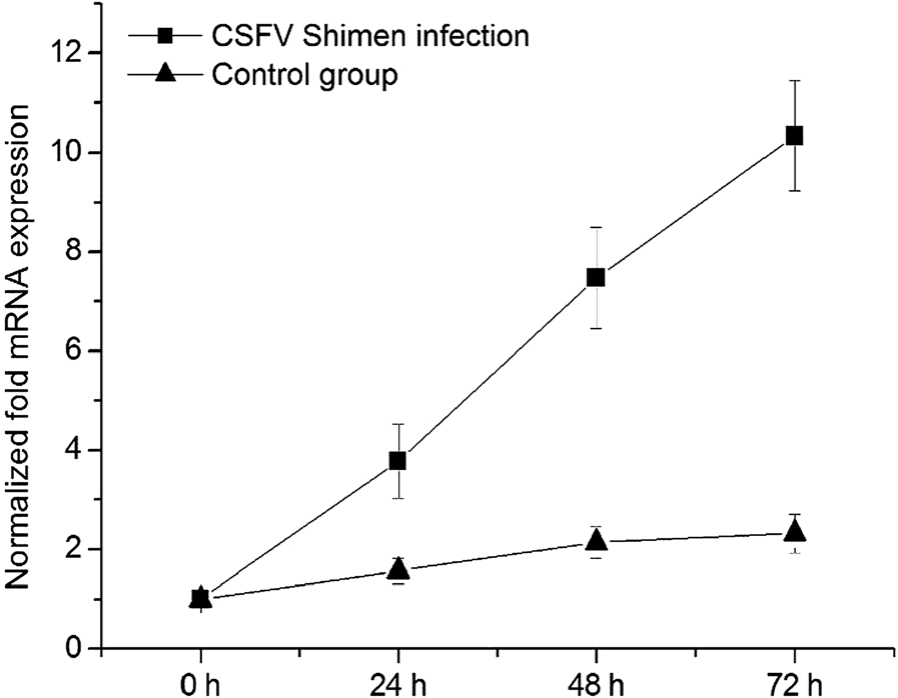
**Analysis of VEGF-C mRNA levels using qPCR assays on SUVECs infected with CSFV Shimen and controls.** qPCR assays was performed to examine the mRNA expression of VEGF-C. β-actin was probed as the loading control. These results are representative of three independent experiments, and data was expressed as mean ± SEM. Control group, mock-infected cells.

The fold increase in VEGF-C mRNA levels were relative to those at 0 h and normalized against β-actin mRNA levels. Infected SUVECs showed significant increases in VEGF-C mRNA levels at 0, 24, 48, and 72 hpi (Figure 3). Control SUVECs showed no increases in VEGF-C mRNA levels over the experimental period.

**Figure 4:**
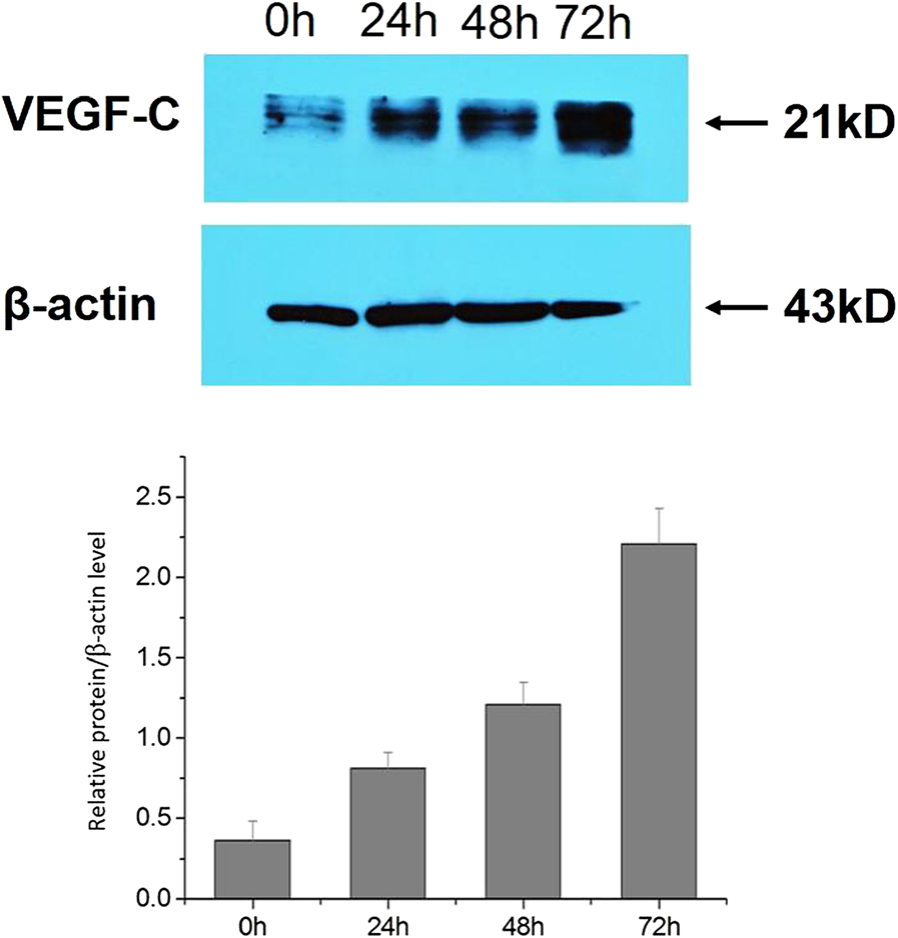
**Effects of CSFV Shimen infection on VEGF-C protein expression.** The experiment was repeated for 3 times with similar results, and data was expressed as mean ± SEM.

VEGF-C protein expression was induced in a time-dependent manner when cells were treated with CSFV Shimen (Figure 4). No significant differences were seen in VEGF-C protein expression among mock-infected cells (data not shown).

## Discussion

In 1989 Leung et al. obtained complementary DNA clones for bovine and human VEGF and reported that VEGF was a secreted angiogenic mitogen [19]. VEGF has been recognized as one of the most important regulatory factors during angiogenesis in human diseases [20]. There are many complex regulatory mechanisms between VEGF family members and a variety of receptors, with the VEGF family, platelet-endothelial-cell adhesion molecule-1, and vascular endothelial-cadherin, integrins jointly competing in the regulation of vascular physiological functions [21]. Although VEGF can promote angiogenesis, over-expression VEGF is damaging to vascular barrier function, and highly increases the permeability of blood vessels. This often results in organ damage in various human diseases [22].

The role of VEGF in veterinary diseases has received little attention compared to that for human-related diseases. A previous study demonstrated that a recombinant orf virus, in which the VEGF-like gene was disrupted, was able to stimulate proliferation of blood vessels in the dermis at the site of infection [23]. This could help understand the infection process of orf viruses and their relationship to proliferative skin lesions in which extensive capillary proliferation and dilation are prominent histological features. Using stem cells, Wang et al. [24] verified that expression of VEGF regulates cardiovascular function in swine models under physiological conditions. Tang et al. [25] found that integrin β3 expression appeared to be significantly up-regulated in SUVECs in conjunction with infection by a virulent CSFV strain.

Currently, deep sequencing technology is used for large-scale analysis of gene expression. In our study, we expected to reveal important genes involved in the host cell response to CSFV Shimen infection using DGE technology. Our findings suggest that VEGF-C was involved in acute infection mechanisms. Besides promoting angiogenesis with VEGF-A, VEGF-C is an important and specific regulatory factor for lymphatic endothelial proliferation and lymphangiogenesis [26]. These processes play important roles in several normal and pathological conditions such as wound healing, inflammation or metastasis formation [27,28].

When acute CSF occurs, CSFV strains can be detected from various tissues and organs from pigs, with the highest viral titers often found in the spleens and lymph nodes [3]. CSFV first invades lymphoid tissues, and then microvascular endothelial cells; therefore initial signs of infection are discolored lymph nodes, with hemorrhaging of the skin observed during the second and third weeks after infection until death [29]. Visible pathological changes are most often observed in the lymph nodes, spleen and kidneys [30]. To validate our DGE analyses, we investigated mRNA and protein levels of VEGF-C.

In conclusion, we showed that VEGF-C was significantly up-regulated as a result of CSFV infection with the Shimen strain. Our results provide a better understanding of the pathogenesis of virulent CSFV infections.

## Competing interests

The authors declare that they have no competing interests.

## Authors’ contributions

PN designed the study, drafted the manuscript and participated in all tests. KG designed primers, participated in collecting samples. RC verified design, participated in collecting samples. WL, ZL and HL participated in testing samples. YZ conceived the study, contributed to the analysis of the results and preparation of revised manuscript versions. All authors read and approved the final manuscript.
